# Pay What You Want! A Pilot Study on Neural Correlates of Voluntary Payments for Music

**DOI:** 10.3389/fpsyg.2016.01023

**Published:** 2016-07-06

**Authors:** Simon Waskow, Sebastian Markett, Christian Montag, Bernd Weber, Peter Trautner, Volkmar Kramarz, Martin Reuter

**Affiliations:** ^1^Department of Psychology, University of BonnBonn, Germany; ^2^Department of Philosophy, University of BonnBonn, Germany; ^3^Center for Economics and Neuroscience, University of BonnBonn, Germany; ^4^Institute of Psychology and Education, Ulm UniversityUlm, Germany; ^5^Key Laboratory for NeuroInformation, Center for Information in Medicine, School of Life Science and Technology, University of Electronic Science and Technology of ChinaChengdu, China; ^6^Department of Epileptology, University Hospital BonnBonn, Germany; ^7^Department of NeuroCognition, Life and Brain Center BonnBonn, Germany; ^8^Department of Sound Studies, University of BonnBonn, Germany

**Keywords:** pay what you want, decision-making and neuroeconomics, music cognition, emotional utility, pricing mechanism

## Abstract

Pay-what-you-want (PWYW) is an alternative pricing mechanism for consumer goods. It describes an exchange situation in which the price for a given good is not set by the seller but freely chosen by the buyer. In recent years, many enterprises have made use of PWYW auctions. The somewhat contra-intuitive success of PWYW has sparked a great deal of behavioral work on economical decision making in PWYW contexts in the past. Empirical studies on the neural basis of PWYW decisions, however, are scarce. In the present paper, we present an experimental protocol to study PWYW decision making while simultaneously acquiring functional magnetic resonance imaging data. Participants have the possibility to buy music either under a traditional “fixed-price” (FP) condition or in a condition that allows them to freely decide on the price. The behavioral data from our experiment replicate previous results on the general feasibility of the PWYW mechanism. On the neural level, we observe distinct differences between the two conditions: In the FP-condition, neural activity in frontal areas during decision-making correlates positively with the participants’ willingness to pay. No such relationship was observed under PWYW conditions in any neural structure. Directly comparing neural activity during PWYW and the FP-condition we observed stronger activity of the lingual gyrus during PWYW decisions. Results demonstrate the usability of our experimental paradigm for future investigations into PWYW decision-making and provides first insights into neural mechanisms during self-determined pricing decisions.

## Introduction

In October 2007, the critically acclaimed band Radiohead provided the most prominent example of the use of Pay-What-You-Want (PWYW) to date, when they offered their fans to pay whatever they wanted for the electronic version of the band’s album *In Rainbows* (Benkler, 2011). In the meantime other bands followed this example in similar ways. Implementations of PWYW are not limited to music distribution but can be found across other economic fields, such as gastronomy and the hotel industry^1^. The somewhat paradoxical success of the PWYW pricing mechanism has triggered a fair amount of scientific research that has demonstrated PWYW’s profitability in various settings (Kim et al., 2009; Regner and Barria, 2009; Belsky et al., 2010; Gneezy et al., 2012; Riener and Traxler, 2012). The question raised by the fact that people pay voluntarily under PWYW conditions is: Why do they do it? Rephrased in economic terms, this means: How do they derive utility from paying a fair amount of money for something that they can get for free? Researchers commonly assert that the buyer’s motivation to pay for a product that they could – in principle – also get for free, is due to the power of social norms, which may outweigh explicit market norms. This, however, is incongruent with the traditional economic view of man as a homo economicus (Persky, 1995; Heyman and Ariely, 2004; Kim et al., 2009). It is assumed, that peoples’ preferences to these social norms are enacted through some kind of *emotional utility*, that can compensate for reduced *monetary utility* (Kim et al., 2009). This view is in line with the dominant neuroeconomic approach to the problem of social preferences (Fehr and Camerer, 2007) and thus, PWYW research may also contribute to new insights in this field.

For the present study, we created a paradigm, in which PWYW payments can be directly compared to payments in a control condition. This control condition was designed to enable us to isolate the unique aspect of PWYW payments, namely that they are given on a voluntary basis, while holding all other factors constant. While other studies compared PWYW payments with a fixed price condition in a between subjects design (Gneezy et al., 2012), the present study is the first to our knowledge to implement PWYW and control condition on the same subjects and in a laboratory environment, which allows a much higher degree of control of confounding variables. We further designed our paradigm to be suitable for fMRI to investigate the neural correlates of PWYW decisions. Our experimental design involved repeated decisions to buy digital music albums. After listening to song snippets (the “listening stage”), participants were asked whether they wanted to obtain this album and how much they were willing to pay (“decision stage”). Crucially, the experimental manipulation included two different contexts at this point. In our control condition, the fixed-price condition (FP condition), participants made a bid on a product with an unknown, randomly determined selling price (the fixed price) and only received the album if their bid was higher than the unknown FP. The PWYW condition in contrast allowed the participant to pay whatever they wanted for the album.

The *FP-condition* required participants to make a rather rational purchase decision, based on only two preferences, one for purchasing a product (music) and an opposing one for keeping the money this product would cost. Multiple studies suggest that the fronto-mesolimbic reward system plays a crucial role in product valuation by responding with increasing activity to increasing valuation (Erk et al., 2002; Knutson et al., 2007; Plassmann et al., 2007). Since the FP (control) condition of our study represents a product-evaluation-decision paradigm we expect a correlation of neural activity in fronto-mesolimbic regions during the *decision stage* (i.e., when confronted with the instruction screen after listening to the song) and the prices paid by the participant.

During the *listening stage*, the neuronal response to our product (i.e., music) has to be taken into account. Converging evidence suggests that the striatal reward system responds to music pleasing to the listener (Blood and Zatorre, 2001; Koelsch et al., 2006; Montag et al., 2011; Salimpoor et al., 2011). Additionally, higher activations in the OFC have been reported in response to attractive products (Erk et al., 2002). We therefore expect that higher striatal and orbito-frontal response to music during the listening stage relates to higher willingness to pay (WTP) in both conditions.

The *PWYW condition* was designed to match the FP condition as closely as possible with the one exception that participants were able to choose any price for the music, including 0.00€. To our knowledge PWYW pricing has only been studied on the behavioral level. In the present study, we will first seek to replicate the findings from these pioneering studies before we proceed to interpret neuronal contrasts between PWYW and FP-condition. In an early study, Kim et al. (2009) delivered evidence for the profitability of PWYW in various real life settings. Their model explains a buyer’s WTP under PWYW conditions (WTP_PWY W_) as a function of his or her internal reference price, which is the price last paid for the same or a similar product. The reference price is assumed to represent a buyer’s WTP for a given product under general (fixed-price) conditions. A higher general WTP will lead to a higher WTP_PWY W_. However, buyers are expected to go for some monetary profit, so their WTP_PWY W_ will be smaller than their general WTP. We therefore expect that buyers pay more than 0,00€ on average in the PWYW condition. We also expect payments in the PWYW to be positively correlated with participants’ general WTP as assessed in the FP-condition and PWYW payments to be smaller than the WTP. Furthermore, we expect that buyers will refuse to buy an album more often in the PWYW condition because of the following rationale: In a fixed price condition, prices are generally assumed to be fixed by the seller and it is not of the buyers’ concern whether the set price is appropriate for the product or not. In voluntary payments as in PWYW, on the contrary, the sole responsibility for determining the price is placed upon the buyer who will not only consider the subjective value of the product (how much they like the album) but also the objective value (e.g., the appropriate price for any music album) and the perspective of the seller (who, e.g., wants to make a living from selling). Thus, voluntary payments may signal a prosocial identity and buyers may tend to avoid to purchase at all when they feel that their WTP might be “too low,” presumably in order to maintain their positive self-image (Gneezy et al., 2012).

By contrasting the two experimental conditions, we are able to isolate the rational aspects of the purchase decision from its social aspects. Two different approaches to the neural correlates of PWYW pricing are conceivable, given the literature. The first approach will focus on the rewarding properties of pro-social behavior. The fronto-mesolimbic reward network represents the key regions that encode social preferences. Following a reward-oriented approach to social preferences (Fehr and Camerer, 2007), it has been shown, that fair actions correlate with greater response of reward related striatal areas in both the beneficiary (Tabibnia et al., 2008) and the donor (Moll et al., 2006). Furthermore, a study by King-Casas et al. (2005) suggests, that activity in the Nucleus accumbens (NAcc) could predict cooperation in a repeated trust game. Thus, when studying the neural correlates of purchase decisions in a PWYW paradigm, we should assume that paying a fair price triggers a response of the buyers reward system. The decision should then results from a trade-off between monetary and non-monetary (an therefore social or emotional) reward (see Kim et al., 2009). The dorsolateral prefrontal cortex (DLPFC) and the ventromedial prefrontal cortex (VMPFC) are likely to be crucially involved in this balancing of competing rewards (Fehr and Camerer, 2007). During the decision stage of the PWYW condition, we therefore expect a correlation between prices and activity in regions of the fronto-mesostriatal network, also activated in the FP condition. However, we expect a stronger activation in PWYW since in the case of high payments, there is an additional source of reward next to the product, namely the reward of having committed a pro-social act. During the listening stage, on the contrary, we expect a less pronounced correlation between prices paid and neural activity in fronto-mesostriatal areas, because in a PWYW situation, prices do not solely depend on product preference, but also on social concerns.

The second approach is less reward based: Although reward is arguably an important aspect of social decision making, a property that *exclusively* applies to social cognition is its relatedness to the intentions of others, namely Theory of Mind (*ToM*; see Amodio and Frith, 2006; Behrens et al., 2009; Young and Dungan, 2012). In the particular context of PWYW, it is vital that the buyer recognizes the sellers intentions, as pointed out by Regner and Barria (2009). Two adjoining regions have repeatedly been associated with tasks that are related to intentions and mental states of others: First, the right temporo-parietal junction (rTPJ; Saxe and Kanwisher, 2003; Saxe and Wexler, 2005), and second, the cortex areas of the superior temporal gyrus and sulcus, which we will refer to as the *STS-Region.* The STS-Region reacts to visually perceived social cues like eye-, head-, and hand-movements (Allison et al., 2000) but is also involved in moral cognition that require high amounts of cognitive control (Borg et al., 2006; Emonds et al., 2011). For the contrast of activity in PWYW and FP-condition *during the decision stage*, we therefore expect that compared to the FP-condition, the PWYW condition should trigger greater activity (a) in frontal regions, associated with social and non-social reward processing, (b) in regions associated with processing of intentions and mental states of others (ToM), like rTPJ and STS, and (c) in regions that respond to emotional content of stimuli, like amygdala and VMPFC.

## Materials and Methods

### Participants

We tested healthy participants (*N* = 25, 12 female, 13 male, mean age *M* = 35.08, *SD* = 17.71), who gave written consent to participate in the study. All analyses were controlled for age and sex. The study was approved by the Ethics Committee of the University Clinics Bonn, Germany (ethics statement: 276/11).

### Stimulus-Material

The stimulus-material consisted of music-albums that were downloaded from Bandcamp.com, an internet platform that allows bands to distribute their music in a variety of pricing formats including PWYW (which, on this site, is called “Name Your Own Price”). In the experiment, we only used albums that were made available by the artists under PWYW conditions or as a free download. The experiment therefore resembles the real life buying conditions for the offered products.

Albums were taken from the Bandcamp.com charts for different musical genres between December 2011 and January 2012. We included music from the following genres: rock, metal, hip hop, country/folk, indie, and pop (Berns et al., 2010). We used the most popular albums that were available via PWYW or free download, and featured at least five songs. Participants were informed that the albums might vary in length. We did not include albums without vocals, compilations with music of different artists, cover- or theme-albums (like Christmas-albums or soundtracks).

We downloaded 14 albums in each of the six genres. At the beginning of the scanning session, participants chose the three genres they liked best. This was done to insure that the general appeal of the songs to the participants would be relatively high. Within each of these genres, seven albums were randomly assigned to both the PWYW- and the FP-condition, which makes for a total of 42 buying decisions, 21 in each condition. All 42 trials were put into a random order. This procedure ensured that the treatment variable “buying condition” is independent of genre, liking or order of the songs.

We selected one song from each album that was played to the participants during the scanning session. This was always the first song, except when the first song was an intro, in which case we used the second one. During scanning, we only presented 30 s excerpts, that would ideally include parts of a verse and a chorus. Note, however, that in order to increase the variance of prices paid, we only allowed participants to bid on an entire album and not on single songs (so they had to infer their buying decision of an album by listening to one sample track).

We emphasized in the instructions, that participants would make real life buying decisions in the experiment and that the artists actually offered their music under PWYW-conditions and would receive the money participants paid. It was not suggested, however, that due to these reasons, there was a moral obligation to pay for the music. After completion of the study, all payments made by participants were transferred to the corresponding artists.

### Buying-Conditions

In the *PWYW-condition*, participants could obtain any album for 0.00€ or any price they chose, up to 10.00€. Whenever participants proposed to pay 0.00€, they were additionally asked, if they wanted to obtain the album for 0.00€ or not, to reduce the ambiguity of this response. In the *FP-condition*, the subjects’ WTP was determined via a classic Becker–DeGroot–Marschak (BDM)-auction (Becker et al., 1964). In this condition, every album had a price that was randomly fixed to any positive value up to 10.00€ and unknown to the participants (the only thing they knew about the price was, that it would never be 0.00€. Therefore, in the FP-condition, a bidding of 0.00€ meant unambiguously that the participant had no interest in obtaining this album). Participants could bid any price they wanted to pay for the album, however, they were informed, that they would only get the album, if their bidding was greater than the randomly fixed price. Participants were informed that in this case, they would buy the record for the randomly fixed price even if they have offered to pay more. Under these circumstances, the participants bidding determines the maximum value of the price he or she may have to pay, and his optimal strategy therefore is to bid his or her true value – the WTP – for any given album.

### Experimental Design

The fMRI-experiment consisted of 42 trials in which subjects had to decide how much to pay for the digital version of a music album (on the behavioral level, we thus studied a total of 1050 buying decisions, distributed over our *N* = 25 participants). Each trial started with a listening stage in which a snippet from a representative song of a given album was played to the participants via headphones. To control for participants’ prior knowledge of the artists and to avoid that the record was already owned by the participant, we obtained a set of records from Bandcamp.com, a website devoted to the distribution of professionally produced records from lesser known amateur bands. Each trial consisted of a listening-stage, a decision-stage and a response-stage. Since a large amount of motion related brain activity was to be expected during this stage, participants were instructed to complete their pricing decision before the beginning of the response-stage and we made no hypotheses about this stage. There were 21 trials in the PWYW- and 21 trials in the FP-condition, that were compared in a within-subject design, with the FP-condition serving as a control condition in which the subjects’ WTP was determined for the present study. Both conditions were identical in every aspect, except for the consequences that the subjects’ pricing-decisions had for their own and the sellers’ pay-off. Participants were initially endowed with a budget of 10.00€, which they could spend fully in every trial. Participants were instructed that this was their money from now on that they were also free to keep the amount partially or entirely for themselves by not spending it all or by not making any purchase. At the end of the experiment, one trial was randomly selected and the transaction was completed depending on the participant’s decision in this trial. By this approach, we made sure that each decision in each trial had the potential to result in a real consequence.

Every album was presented only once in the course of the experiment, to ensure a novelty aspect of the product. This means, however, that we can only compare average prices between the two buying conditions. Stimulus timing was 30 s for the listening stage, followed by a 3 s instruction slide indicating the condition (PWYW or Fixed-Price) and a 5 s time window that allowed participants to think about the price they were willing to pay, which makes for a total of 8 s for the decision stage. This stage was followed by the input stage, in which participants were asked to enter the amount they had decided on (the stages of a prototypical trial are shown in **Table 1**). The input stage lasted as long as it took the participant to enter the price in each trial and thus served as a temporal jitter. A similar timing structure was used in Knutson et al. (2007).

**Table 1:** Stages of each trial.

Stage	Listening	Decision		Response
Duration	30 s	3 s	5 s	Variable
Display/Stimulus	Album-Cover + Music	PWYW	PWYW Think about your price!	5.00€

*The table shows a trial of the PWYW condition. Condition names were not abbreviated but spelled out during the actual experiment. In the FP-condition, the PWYW display was replaced by “Fixed Price.”*

### Image Acquisition

fMRI data was recorded on a 1.5T Scanner (Avanto, Siemens, Erlangen, Germany) with a standard 8 channel Siemens head coil. We collected about 800–1000 T2^∗^-weighted, gradient echo EPI-scans, depending on how much time participants needed to type in their prices. The following parameters were used: 31 slices per volume; slice thickness: 3 mm; inter-slice gap: 0.3 mm; matrix size: 64 × 64; echo time: 45 ms; repetition time: 2500 ms; flip angle: 90°. Structural images were obtained by collecting 160 T1-weighted volumes (repetition time: 1660 ms; echo time: 3.09 ms; flip angel: 15°; slice thickness: 1 mm).

### Image Processing

Functional images were preprocessed using SPM8. Preprocessing included the following steps in the given order: (a) slice timing (b) realignment for motion correction (c) co-registration with the high resolution spatial images (d) spatial normalization using SPM’s unified segmentation routine and (e) smoothing with a Gaussian spatial filter with 8 mm full width at half maximum.

Preprocessed data were analyzed using a general linear model, fitted using SPM8’s canonical hemodynamic response function and a high pass filter of 128 seconds. Each stage of the experiment (listening, decision, and input) in each condition (PWYW and FP) was modeled as a separate regressor (i.e., six orthogonal regressors). We also included additional regressors parametrically modulating the listening- and decision-stage by the prices participants paid on these trials. The modulators were included to investigate linear dependencies between brain activity and prices paid. On each trial in either condition (PWYW and FP), participants had the option to indicate that they were not willing to pay any money at all. In the FP-condition, this would have inevitably revoked the chance to obtain the album at all. Because it cannot be ruled out that the decision to not buy an album is qualitatively different from paying even a little sum, we decided to exclude trials with 0.00€ payments and model them as separate regressors. In five participants (three men and two women), this led to a reduction of valid experimental trials by more than 50%. We therefore excluded these participants from the analysis of imaging data. In the PWYW-condition, however, it was possible to obtain an album for free by entering a price of 0€. To distinguish this situation from occasions where the participant rejected the album even if it was free, we interrogated participants each time they entered 0.00€ if they wanted to obtain the album for free. This, however, did only occur five times across all participants and trials. All other trials, in which participants entered 0.00€ were modeled with separate regressors as in the FP-condition. Six motion parameters were added as regressors of no interest to the model to account for residual head motion not corrected during preprocessing.

Contrast estimates from the GLM analyses were submitted to a second-level analysis that treated subjects as random effects and modeled participants age and sex as covariates of no interest. Resulting statistical parametric maps were initially thresholded at *p* < 0.001 and then corrected for the family-wise error at the cluster level to keep the probability of false-positive results beneath *p* < 0.05 at the whole brain level.

## Results

We will start with presenting behavioral results to demonstrate the effectiveness of our experimental manipulation and the consistency of our results with previous work. We will then present the neuroimaging results from the FP-condition that reflect neural correlates of a “traditional” (“rational”) exchange situation. After this, we present the neuroimaging results regarding the PWYW condition, and differences in brain activation between the two buying conditions. We will conclude the results section by a meta-analysis to provide a solid ground for the interpretation of our main finding.

### Behavioral Results

The analyses of the behavioral data include the entire sample of 25 participants. The same results, however, were obtained for the reduced sample of 20 participants who were included in the imaging analyses. All analyses are based on four descriptive measures: The average prices paid for an album in the PWYW and FP-condition, their difference (priceFP – pricePWYW) and their ratio (pricePWYW/priceFP). These measures were computed in two ways: uncorrected measures comprise the price inputs of all trials. As not all trials actually involved the purchase of an album in all participants (like an input of “0.00€” in the FP-condition or input of 0.00€ and answering “no” to the question of whether they wanted to have the album for free in the PWYW condition), we corrected the measures for these “non-transactions” by excluding these trials from the analyses. Descriptive statistics for all four measures in their uncorrected and uncorrected versions are presented in **Table 2**.

**Table 2:** Descriptive statistics of corrected and uncorrected prices (means in €).

	Corrected	Uncorrected
Price PWYW (WTP_PWY W_)	3.10 (*SD* = 1.31)	2.37 (*SD* = 1.26)
Price FP (WTP)	3.49 (*SD* = 1.06)	2.96 (*SD* = 1.27)
Difference FP-PWYW	0.39 (*SD* = 0.89€)	0.59 (*SD* = 0.86)
Quotient PWYW/FP	0.87 (*SD* = 0.29)	0.83 (*SD* = 0.43)

In line with our hypotheses and previous behavioral findings, participants took advantage of the PWYW offer and paid less in this condition. However, prices paid in the PWYW condition were significantly higher than 0.00€ [*t*(24) = 11.85, *p* < 0.001 for corrected, *t*(24) = 9.43, *p* < 0.001 for uncorrected prices], replicating previous results that demonstrated the general feasibility of the PWYW pricing system. Even though prices paid in the FP and in the PWYW condition were highly correlated (*r* = 0.73; *p* < 0.001 for the corrected means of both buying conditions, *r* = 0.77, *p* < 0.001 for uncorrected means) reflecting that participants were guided by their general WTP when determining how much they would pay in the PWYW condition (Kim et al., 2009), there was still a significant difference in the amounts participants paid in the two conditions [*t*(24) = -2.15; *p* = 0.042 for the corrected, *t*(24) = -3.41; *p* = 0.002 for the uncorrected measures]. In line with results from Gneezy et al. (2012), participants in our study refused to buy an album more often in the PWYW condition (that is, out of 525 trials in each condition, participants decided 128 times that they would not buy an album, not even for a price of 0.00€, in the PWYW condition, but only refrained from a purchase 91 times in the FP condition; *p* < 0.01; χ^2^ = 7.90; *df* = 1; *N* = 1050). This could be interpreted as a tendency to rather not buy the record before paying a price that might be “too low.” In sum, behavioral results indicate that participants distinguished between the two conditions as expected from the previous literature, and adjusted their decisions accordingly (Kim et al., 2009; Regner and Barria, 2009; Riener and Traxler, 2012). PWYW payment was the only variable that showed associations with sex and age: Men payed higher prices than women, but only for the corrected prices [*M* = 3.66, *SD* = 1.18 vs. *M* = 2.49, *SD* = 1.20, *t*(23) = 2.454, *p* = 0.022 for corrected prices] and age was positively correlated with the corrected PWYW prices (non-parametric *r* = 0.579, *p* = 0.002 for corrected and non-parametric *r* = 0.44, *p* = 0.028 for uncorrected means). Controlling for age and sex, however, did not affect the behavioral results.

### Imaging Results

#### Fixed-Price

**FIGURE 1:**
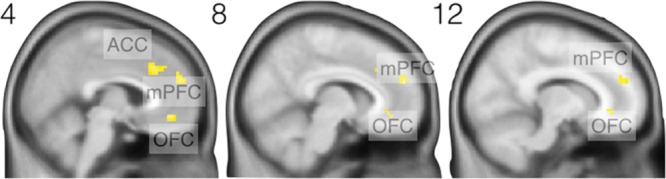
**Frontal brain regions that show a positive correlation between prices paid under FP-conditions and neural activity in the decision stage.** Numbers refer to MNI coordinates of the sagittal slices.

Our first analysis focused on the parametrically modulated regressor of the *decision stage*, to investigate whether participants’ WTP was reflected in neural activity at this stage. **Figure 1** shows the statistical parametric map of the respective second level analysis: Neural activity in three clusters correlated positively with the prices paid in the fixed price condition: One cluster in the orbitofrontal cortex (OFC; peak coordinate *x* = 0, *y* = 41, *z* = -11, *Z* = 4.35, *p* < 0.05, corrected, *k* = 40 voxels), one cluster in medial prefrontal cortex (mPFC; *x* = 9, *y* = 53, *z* = 22, *Z* = 4.00, *p* < 0.05, corrected) *k* = 38 voxels, and one cluster in the anterior cingulate (ACC; *x* = 0, *y* = 29, *z* = 31, *Z* = 3.78, *p* < 0.05, corrected *k* = 55 voxels). These results indicate that brain regions implicated in reward processing encode participants’ WTP. Corresponding results can be found in Knutson et al. (2007) and Plassmann et al. (2007).

The participants’ WTP should be related to their preference for the product they are about to purchase. It is likely that this product preference emerges during the *listening stage* while the participant makes first contact with the music. We therefore investigated next, whether a similar linear relationship between WTP and neural activity existed during the listening stage. No significant results were obtained at the whole brain level. A more focused search around the peak coordinates of the significant clusters from the previous analysis on the linear dependencies between participants’ WTP and neural activity during the decision stage, however, revealed a similar relationship between WTP and neural activity in the orbitofrontal cortex during the listening stage as well (*Z* = 4.25, *p* < 0.05, small-volume corrected in a 10-mm-radius sphere around *x* = 0, *y* = 41, *z* = -11). This result is in line with previous findings of a response of the OFC to product-attractiveness (Erk et al., 2002; Plassmann et al., 2007). No results were obtained when small-volume-corrected searches were conducted around the clusters in the medial frontal cortex and the ACC.

#### Pay-What-You-Want

Our analysis of neural activity during the different stages in the FP-condition has revealed a linear relationship between participants’ WTP and neural activity in brain areas involved in reward-processing. Next, we investigated whether a similar relationship existed in the PWYW condition. Focusing on the linear relationship between prices paid and neural activity in the *decision-stage*, we were not able to find a significant relationship in any brain regions, neither at the whole brain level, nor when focusing on the peak coordinates found in the fixed price condition, nor when lowering the threshold to *p* < 0.001, uncorrected. Similarly, no relationship between prices paid and neural activity was detected during the *listening stage* of the PWYW condition. While neural activity in reward-related brain areas appears to be predictive for the WTP under traditional fixed-price exchange conditions, this type of relationship does not seem to exist under PWYW conditions. This is remarkable, since the two conditions did only differ with respect to the pricing mechanism, and our finding that prices paid in both conditions were correlated, indicates that participants’ pricing decisions in the PWYW condition were not random.

**FIGURE 2:**
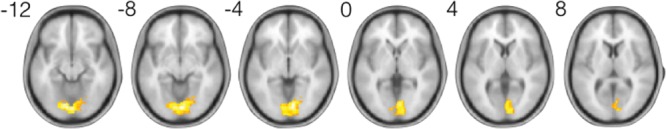
**Increased activity in the lingual gyrus during PWYW decisions in comparison to fixed-price conditions.** Numbers refer to MNI coordinates of the transverse slices.

We next investigated *differences* in neural activity between the two conditions by directly contrasting the two pricing conditions during the decision stage, the time when participants were first confronted with the pricing context in this experimental trial. Note that this contrast did not make use of parametrically modulated regressors. At this stage, we found increased neural activity when participants were confronted with the PWYW condition, compared to the FP context, in the occipital lobe, peaking in the lingual gyrus (see **Figure 2**, *x* = 3, *y* = -85, *z* = -8, *Z* = 4.85, *k* = 389 voxels, *p* < 0.05, corrected). No results were obtained for the reverse contrast (FP > PWYW) at the selected threshold.

### Meta-Analysis

In contrast to reward-, risk-, and higher cognition-related brain areas, the lingual gyrus has not received much attention in the neuroeconomic literature. Together with the behavioral results, our experimental design, however, suggests an implication of this brain area in PWYW decision making. We conducted an automated meta-analysis within the NeuroSynth-framework (Yarkoni et al., 2011) to obtain a quantitative reverse inference on the peak activation coordinate in the lingual gyrus. We queried the NeuroSynth database^2^ that encompassed 413,429 activation coordinates from 11,406 studies in August 2015 to obtain information on the probability that studies contained a certain search term given activation at this specific location. The posterior probability measure from the NeuroSynth database is a measure for selective activation of a brain region and can allow for inferences on psychological states from brain imaging results. As the posterior probabilities derived from NeuroSynth are not corrected for uncertainty, we report only results with a significant *z*-statistic.

While all main associations were reported for visual processing (*z* = 4.57, posterior probability 0.68), visual attention (*z* = 4.11, posterior probability 0.82), or simply anatomical location, one search result suggested an implication of this region in emotional information processing (*z* = 4.1, posterior probability 0.84). In a next step, we queried the database for two additional coordinates that were local activation maxima within the activated cluster obtained from the PWYW > FP contrast. According to NeuroSynth, the local maximum at MNI (-9, -82, -11) is associated with the terms “memory” (*z* = 3.55, posterior probability 0.64), “autobiographical” (*z* = 4.84, posterior probability 0.83), and “retrieved” (*z* = 4.03, posterior probability 0.83). The second local maximum at MNI (9, -88, 4) was only associated with search terms related to visual processing.

## Discussion

The present study’s experimental paradigm was designed to elucidate the relationship between neural activity and participants’ willingness-to-pay under two different pricing regimes: a FP-condition resembling a traditional exchange between a seller and a consumer, and a PWYW condition that resembled the fixed-priced condition in every way, with the only exemption that in the PWYW condition, consumers were given the option to pay any price they wanted. On the behavioral level, we replicated previous findings on the feasibility of the PWYW approach: Even though participants decided to pay significantly less when the pricing decision was in their hands, they still offered to pay amounts significantly greater than zero. Also, as in the study of Gneezy et al. (2012), participants refused to buy an album more often in the PWYW than in the FP-condition. On the neural level, we found supporting evidence for our hypothesis on the relationship between mesolimbic-frontal activity and willingness-to-pay. Such a relationship, however, was only present in the fixed price condition. In the following, we will discuss this finding and the absence of such a relationship in the PWYW condition and seek possible explanations for apparent differences in neural activation between the two pricing conditions.

### FP-Condition

During the *decision stage*, we found three areas in which activity was positively correlated with prices paid on a trial basis: the mPFC, the OFC, and the ACC. All of these areas are known candidates for higher cognitive function and decision making in economic contexts. Our finding in the MFC is in line with results from a study of Knutson et al. (2007; -4, 59, -3 and 4, 46, -6), who found this region to respond to price information and to be more active in cases in which participants found the price to be appropriate and purchased the presented product.

For our result from the OFC, we find corresponding evidence in Plassmann et al. (2007) who could show, that the medial OFC (as well as the right DLPFC) correlated with participants’ WTP (similar results were obtained by Erk et al., 2002). While Plassmann et al. (2007) presented primary rewards such as food stimuli to hungry participants, our results show that the mOFC also reacts to others rewards such as music. Again, this is in line with the results from Erk et al. (2002) who used pictures of more or less attractive cars as stimulus material. The ACC has also been implicated in decision making, especially with respect to action selection, as discussed by Rushworth et al. (2007). The ACC and its interconnectivity with the mPFC has also been positioned in a framework of evaluation, appraisal, and conflict-resolution (Etkin et al., 2011). Our present design does not allow to disentangle reward- and conflict-based accounts. This will be an interesting endeavor for future research.

During the *listening stage*, we observed a similar relationship between neural activity and WTP at the same location in OFC as during the decision stage. Previous research on the neuronal response to music has primarily focused on a different structure during music reception by showing that a positive response to music correlates with higher activity in the Striatum. However, these studies were either based on the presentation of reported favorites of the participants (Blood and Zatorre, 2001; Montag et al., 2011; Salimpoor et al., 2011) or contrasted “pleasant” with heavily dissonant music (Koelsch et al., 2006). In contrast, our study made use of musical material that was previously unknown to the participants and also generally pleasing since it consisted of the top albums of the Bandcamp.com charts for each of our musical categories.

We found a correlation between activity in the OFC during music presentation with the prices later paid. Especially, since this ROI corresponds precisely to the cluster that also correlates with the price during the decision stage, we should assume that the OFC is involved in product (music) valuation. This is consistent with the repeated findings of striatal activity in response to pleasant music, due to strong anatomic connections between striatal areas and the OFC (Plassmann et al., 2007). We should note that this correlation has predictive value, since the participants in our study were only informed about the condition under which they had to decide their price *after* the listening stage. Further, because this correlation is calculated by a parametric modulated regressor, our result is sensitive to the shared variance of price and neural activity on the individual level.

### PWYW Condition

Even though the behavioral data on PWYW decisions was in line with the previous literature, we found no correlations between BOLD-signal and prices paid in the PWYW condition. Even though null-findings are difficult to interpret, we can conclude that the straight forward translation of product preference into prices that we found in the FP-condition does not exist in the PWYW condition in the same way. The high correlation between the mean prices in the two buying conditions across participants suggest some systematic behind the pricing decisions, which should be reflected in neural activation data. A possible explanation for the apparent null finding might therefore be a higher degree of between-subject variability in the decision making mechanisms under PWYW-conditions that preclude robust activations at the group level. A possible avenue for future studies might therefore be the application of multivoxel pattern classification analyses that have been shown to decode signals from sub-threshold activation data on the single-subject level (Riggall and Postle, 2012).

Instead of a correlation between neural activity and prices paid, or significant activation in Theory of Mind related areas like the STS-Region, we found an unexpected, but highly robust contrast of activity between the two buying conditions in the Lingual Gyrus during the decision stage. How can we explain this result? The lingual gyrus is part of the secondary visual cortex. At this point, it should be stressed once more, that our two experimental conditions only differed in respect to which condition-name was presented and in which options this resulted for the participants pricing decision. The condition-name therefore served as a visual cue for different sets of options to be considered in determining the price, and considering this, it is not surprising, that visual discrimination plays a crucial role in our setting. Hence, we find that in both conditions, activity of the occipital cortex is higher than baseline. This effect is significantly greater for the PWYW condition and comprises a bilateral cluster of 389 voxels (see **Figure 2**, *x* = 3, *y* = -85, *z* = -8, *Z* = 4.85, *k* = 389 voxels, *p* < 0.05, corrected). It is unlikely that the activation difference is a merely perceptual in response to the cue. We will argue that the activation difference reflects an affective response to the indication of different pricing schemes. Previous findings concerning the lingual gyrus can support this interpretation.

Adolphs (2002) has argued that the visual cortex plays a role in the early processing of emotional stimuli and the lingual gyrus has repeatedly been associated with reaction to emotionally relevant stimuli (Taylor et al., 1998; Critchley et al., 2000b; Moll et al., 2002; Rilling et al., 2008; Fusar-Poli et al., 2009; Premkumar et al., 2012).

A meta-analysis of fMRI studies (Fusar-Poli et al., 2009) was able to show that areas of the visual cortex like lingual, inferior occipital and fusiform gyrus react to the emotional expression of faces. Furthermore, they showed, that the lingual gyrus reacted more strongly to sad than to neutral faces and especially played a role in the implicit processing of facial expressions. In a study, in which participants had to discriminate between scenes showing social acceptance or rejection, Premkumar et al. (2012) found, that the lingual gyrus reacted more strongly to rejection, which is in line with findings of Rilling et al. (2008). The authors conclude that the lingual gyrus plays a role in discriminating different qualities of social information. In addition, participants in this study, who *reported* lower emotional arousal in response to the scenes displayed, showed a more pronounced contrast in the lingual gyrus than others, suggesting a connection of this area to an important peripheral marker of emotional experience. The possible involvement of the lingual gyrus in emotional information processing is further corroborated by our own NeuroSynth analysis which showed a possible association with our peak activation location and affective processing. It should be noted, that the point of peak activation of the lingual gyrus within the 8 s of the decision-stage is unknown. A minimal interpretation of our findings could be, that the norm-related PWYW condition requires a “special attention,” because decisions under this condition may be relevant for emotional regulation, which is not the case under FP conditions.

We have to ask, however, why other areas associated with emotional processing, like the amygdala and VMPFC, don’t show up in our contrast of PWYW and FP-condition. Visual cortex and STS-region are both connected to these areas, and especially the amygdala is believed to influence processing in the visual cortex and the STS-region via feedback projections (Allison et al., 2000; Critchley et al., 2000a). Our findings in the visual cortex may support the assumption of early discrimination between stimuli with and without social components, possibly before they are assigned an emotional valence. The absence of any straightforward correlations between BOLD-signal in the PWYW condition, and the amount of money paid to the seller could suggest that after an early discrimination between the two buying conditions, participants used different strategies to determine their prices in the PWYW condition. We should still assume that these are influenced by the early discrimination.

Finally, the possibilities that our PWYW paradigm offers for future research on social and economic decision making should be noted. The paradigm implements two frames, in which decisions can take place, one organized primarily by rational considerations (the explicit rules of the market), and one that additionally includes the consideration of implicit social norms. The paradigm manages to implement this contrast while maintaining a degree of external validity, which is unusually high for fMRI experiments: A usual purchase of music via Bandcamp.com or other internet platforms does in fact happen via a screen (which is not the case for chocolate bars) and money is (or is not) paid to a real artist, who is only present in form of the music and cover information on the platform. Our design offers a contrast of two conditions which differ only in TOM aspects, except for one cue about the pricing condition. Potential uses of this paradigm should be investigated further: First, cross-modality validation, for example by replacing the visual cue about the buying condition with an auditory one, should yield insight in the role of the lingual gyrus. A combination of fMRI and measurements of electrodermal activity as in Critchley et al. (2000b), could also be considered, to gain insights into arousal processes and the embodiment of decision process as proposed in the somatic marker hypothesis. In the present study, we did not obtain independent measures of preference for each song. This could be helpful, however, to achieve clearer results on neuronal activity in the PWYW condition. Also, it should be investigated, how exactly the “socialness” of the PWYW condition is perceived, for example by distinguishing two conditions in which the degree to which social information is made salient varies. Personality should be taken into account as well to account for between-participant variation in WTP under both conditions. Interesting traits would be those that either relate to social behavior such as “cooperativeness” (Cloninger et al., 1993) or “openness to experience” (Costa and McCrae, 1992) which could underly the willingness to explore unusual or innovative ideas such as a PWYW system.

To maintain statistical power for the comparison of neuronal activity in different subgroups, a bigger sample and/or more experimental trials would be needed. A point that warrants discussion is the use of an BDM-auction in the fixed-price conditions. While this approach has been popularized by auction enterprises such as ebay.com, it differs from the usual experience of buyers in a traditional shop where tags provide information about prices. The BDM-auction, however, is usually used in neuroeconomic research to obtain a direct and bias free measure for participants’ WTP (Weber et al., 2007) which was the desired goal in our experiment. Another concern in our study is the amount of trials in both conditions. We tried to include as many trials as possible to obtain sufficient statistical power but at the same time tried to limit the number of trials to maintain enough ecological validity as buyers in real exchange situations tend not to make too many consecutive buying decisions. During analysis, we excluded trials in which no transaction took place as they might have been qualitatively different from actual transactions. Even though, we excluded participants with too many dropped trials, we cannot rule out entirely that the null result regarding the correlation between neural activity and prices payed in the PWYW condition resulted from a reduced statistical power due to unequal trial numbers in both conditions. Trials in the PWYW condition were excluded when participants bid 0.00€ and indicted that they did not want to obtain the album for free in response to a subsequent question. While this was effective to distinguish between qualitatively different decisions that both resulted in a 0.00€ bid, future studies may want to make use of a “reject” button that allows buyers to reject undesired albums right away.

To our knowledge the present study is the first neuroeconomic imaging study to investigate neural correlates of the PWYW pricing system. PWYW has recently received a lot of attention in the behavioral literature, presumably sparked by reports on its surprising success in the marketing of music albums (Benkler, 2011). Alongside first evidence on neural underpinnings of PWYW buying decisions, we present an experimental design that allows the study of different pricing mechanisms in a neuroimaging setting. We would like to encourage further studies on this topic, for instance with machine learning techniques that aim at the prediction of PWYW decisions from multivariate patterns in neural activity. In general, our paradigm is also suitable for the study of other products than music albums. A replication of the present finding using different products/stimuli would provide further valuable insights.

## Author Contributions

SW, SM, MR, CM, and BW designed the research; SW and SM conducted the experiment, analyzed the data, and wrote the manuscript; VK, PT provided protocols and technical advise; MR, CM, BW, VK, and PT edited the manuscript.

## Conflict of Interest Statement

The authors declare that the research was conducted in the absence of any commercial or financial relationships that could be construed as a potential conflict of interest.

## Footnotes

1 www.pay-what-you-want.net

2 www.neurosynth.org
